# The Latest about Bacteria

**Published:** 1882-12

**Authors:** 


### THE LATEST ABOUT BACTERIA.

This time it comes from America.

Dr. Formad, of Philadelphia, has made some experiments, from which he is led to believe that, contrary to the generally accepted view, bacteria are not the *per se* of disease, but are merely the vehicle of contagion, the means by which the poison of certain diseases is carried from one organism to another. He has found the tubercle bacilli of Koch, or, at least, bodies identical with them, in the sputa of non-phtisical patients.

He has taken matter infested with the diphtheritic micrococci (first demonstrated by Professor Wood and himself), and has succeeded in producing the disease in animals, while micrococci from the very same specimen, *after a thorough washing in plain water*, were perfectly harmless.

He believes that bacteria exist in all nature, and that, even when charged with the elements of disease, they cannot produce the disease unless they find a resting place in some body that is, on account of some unexplained conditions, a suitable pasture for the growth and development of the particular disease.

This view, which seems to be a very rational one, has two important practical bearings.

In the first place, it teaches us very forcibly the value of water as a disinfectant, and in the second place, it lends much additional force to the idea that infectious diseases are due to chemical influenees, which theory, if demonstrated, will do much toward increasing the potency of our therapeutic resources.

If these bacteria carry certain elements that are capable of producing disease, it is not unreasonable to hope that chemistry will step in and tell us the chemical nature of these poisons ; from this standpoint it will be but a step to name the chemical that will prove the antidote, and by this process can we arrive at the most rational treatment of infectious diseases. Dr. Formad promises to have more to say on this subject, and we look forward to his researches with interest, for it would seem that he is on the right track.—*Med. and Surg. Rep.*

